# Play fighting social networks do not predict injuries from later aggression

**DOI:** 10.1038/s41598-020-72477-7

**Published:** 2020-09-23

**Authors:** Simon P. Turner, Jennifer E. Weller, Irene Camerlink, Gareth Arnott, Taegyu Choi, Andrea Doeschl-Wilson, Marianne Farish, Simone Foister

**Affiliations:** 1 Animal and Veterinary Sciences, SRUC, Roslin Institute Building, Easter Bush, Midlothian, UK; 2 School of Biological Sciences, Queen’s University Belfast, Belfast, Northern Ireland UK; 3 Institute of Genetics and Animal Biotechnology, Polish Academy of Sciences, Jastrzebiec, Magdalenka, Poland; 4 The Roslin Institute and Royal (Dick) School of Veterinary Studies, University of Edinburgh, Edinburgh, Midlothian UK; 5 Present Address: Agri-Epicentre, Edinburgh Technopole, Penicuik, EH26 0BA UK; 6 Present Address: Department of Comparative Pathobiology, Purdue University, West Lafayette, IN USA

**Keywords:** Developmental biology, Ecology, Psychology

## Abstract

Early play fighting mimics later aggression in many species, and may, therefore, be expected to reduce costs from later aggressive interactions. Using social network analysis (SNA) the effect of a central play fighting network position on later skin lesions from aggression was assessed in domestic pigs. Piglets (n = 263) were kept in litter groups or socialised pre-weaning with another litter to enhance play fighting experience. Play fighting was recorded for 1.5 h per day over 6 days pre-weaning. Play fighting network centrality was quantified using measures of individual network position and entire network structure (degree, eigenvector, betweenness, clustering coefficient). Skin lesions from aggression were counted after a dyadic contest and at 24 h and 3 weeks following group mixing. Pigs with play fighting interactions with many partners experienced fewer lesions from the dyadic contest (in-degree, p = 0.01) and tended to received fewer lesions 3 weeks after group mixing (degree, p = 0.088) but no other play fighting centrality measures affected the number of lesions at any point. The benefits of play fighting were therefore limited to specific aggressive social contexts. The tendency of socialised piglets to play fight with non-littermates did not affect subsequent lesions. We advocate the use of SNA over approaches that only consider dyadic interactions to further our understanding of the influence of early social group interactions on later life experience.

## Introduction

The young of many species engage in energetically demanding play fighting that mimics, at least in part, behaviours performed during adult aggression. Typically play fighting occurs without injury and opponents may cooperate in allowing role reversals (characterised more fully by Smith (1997)^1^ and Burghardt (2005)^2^). Many hypotheses exist to explain social play, of which play fighting is a major component. Proposed benefits include improved motor coordination (motor training hypothesis^3^), social cognitive development (e.g. self-assessment hypothesis^4^), and emotional control (e.g. training for the unexpected hypothesis^5,6^). It has been proposed or assumed that play fighting improves the fitness benefits from later-life true aggressive interactions (e.g.^7–12^). If this is correct, it would be expected that engagement in play fighting would help to reduce the costs or increase the benefits of later-life contests. However, where this has been tested, studies conflict in whether engagement in play fighting alters the outcomes of aggressive interactions (e.g. Syrian golden hamsters, *Mesocricetus auratus*^13^; meerkats, *Suricata suricatta*^14^, yellow-bellied marmots, *Marmota flaviventris*^15^; domestic pigs, *Sus scrofa domesticus*^16^). The conflict between these studies might represent genuine species, sex or age differences in the function of play fighting. Alternatively, it may partly result from discrepancies in the recording, summarising or analysis of play fighting interactions or the involvement in, and outcomes from, later-life real aggression.

Play fighting includes elements of both competition and cooperation and the emphasis placed on each depends upon the species^17^. Play fighting involves frequent use of self-handicapping postures and tactics and, in many species, can mimic aspects and body targets of adult behaviours that are not associated with real fighting (e.g. mating and hunting)^6^. Inclusion of these elements in play fighting ought to weaken its specific benefits for later aggressive contest resolution. However, Pellis and Pellis (2016, 2017)^6,18^ have shown that play fighting in various species of pigs of the family Suidae lacks self-handicapping. In these animals, play fighting vigorously and faithfully mimics adult fighting except in the scarcity of injuries, the honouring of submissive signals following defeat and the willingness of defeated animals to stimulate new bouts of interaction. Indeed, Šilerová et al. (2010)^19^ have concluded that playing and fighting may form a single continuum in pigs. These rules of play fighting ought to maximise benefits of play for later contest behaviour and makes pigs an ideal subject with which to identify these benefits. Pigs engage in much social play from the age of 3–5 days^12^, have dominance hierarchies in wild matrilineal social groups and show intense male competition during the breeding season^20–22^. Furthermore, aggressive interactions can be readily staged in captivity and have quantifiable costs (e.g. superficial skin lesions as a consequence of being bitten^23^). Furthermore, this species has proven a useful model for studies on fundamental aspects of aggression^24,25^ and play^16,26^. Here we test whether engagement in play fighting in domestic piglets affects later costs (reflected in skin lesions) from aggressive contact with unfamiliar pigs.

Previous studies have quantified engagement in play fighting as the total frequency or duration of play interactions or the number of play partners that an animal engaged with. This approach takes no account of the identity of the social partners but many studies show that dyads do not interact at random (e.g.^27–30^). Furthermore, past efforts to quantify play assumed that engagement with each potential play partner would yield the same benefit, which is unlikely to be true. At the simplest level, choosing to engage with a partner who has never experienced play fighting is likely to lead to different benefits than choosing a partner who is highly experienced. The current study seeks to quantify play engagement through the more sophisticated lens of social network analysis and examine its relationship with later aggression. Social network analysis does not suffer from the weakness of earlier methods of quantifying social experience that assumed that dyads interact independently of their wider social group^31^.

Social network analysis is a powerful tool that considers interdependencies within a group (the ‘friends of friends’) beyond the dyad level^31^. Positions of centrality of an individual within its entire network can be mathematically quantified and can affect fitness (e.g.^32–35^). In the context of social play, social network analysis can quantify novel characteristics such as how central or peripheral an individual is in the play fighting network, whether its position is key in the group (e.g. operating as the gatekeeper between play subgroups) and the extent to which its play partners are experienced in play. Despite these benefits, to our knowledge no study has examined how position in a play fighting network affects later outcomes of aggressive behaviour.

Using social network analysis, we test whether centrality of piglets in their play fighting network predicts the number of skin lesions resulting from a dyadic contest between two unfamiliar pigs several weeks later and thereafter in the highly dynamic scenario of formation of a new social group. We sought to maximise variation in play fighting opportunities by allowing some litters the opportunity to interact with an adjacent litter before weaning, in a process termed socialisation^36^, with others maintained in their natal group. We make two specific predictions. Firstly, piglets that are central in their play fighting network will experience fewer injuries following a staged dyadic contest and later formation of a new social group (Prediction 1). This follows from a rationale that those located centrally in the play fighting network will have gained greater social or physical skills for later life aggression. Secondly, we predict that piglets that play fight with non-littermates when given the opportunity of early-life socialisation will receive fewer injuries from later life aggression than those which play preferentially with littermates (Prediction 2).

## Methods

### Ethical note

The data were derived from a larger study conducted for other purposes at the SRUC Pig Research Centre. The study was approved by the UK Home Office (Project licence PPL60/4330) and SRUC ethical review committee (application ED RP 04-2014) and all methods were carried out in accordance with relevant guidelines and regulations. No animal required veterinary attention as a consequence of any experimental procedures.

### Experimental design and housing

**Figure 1:**
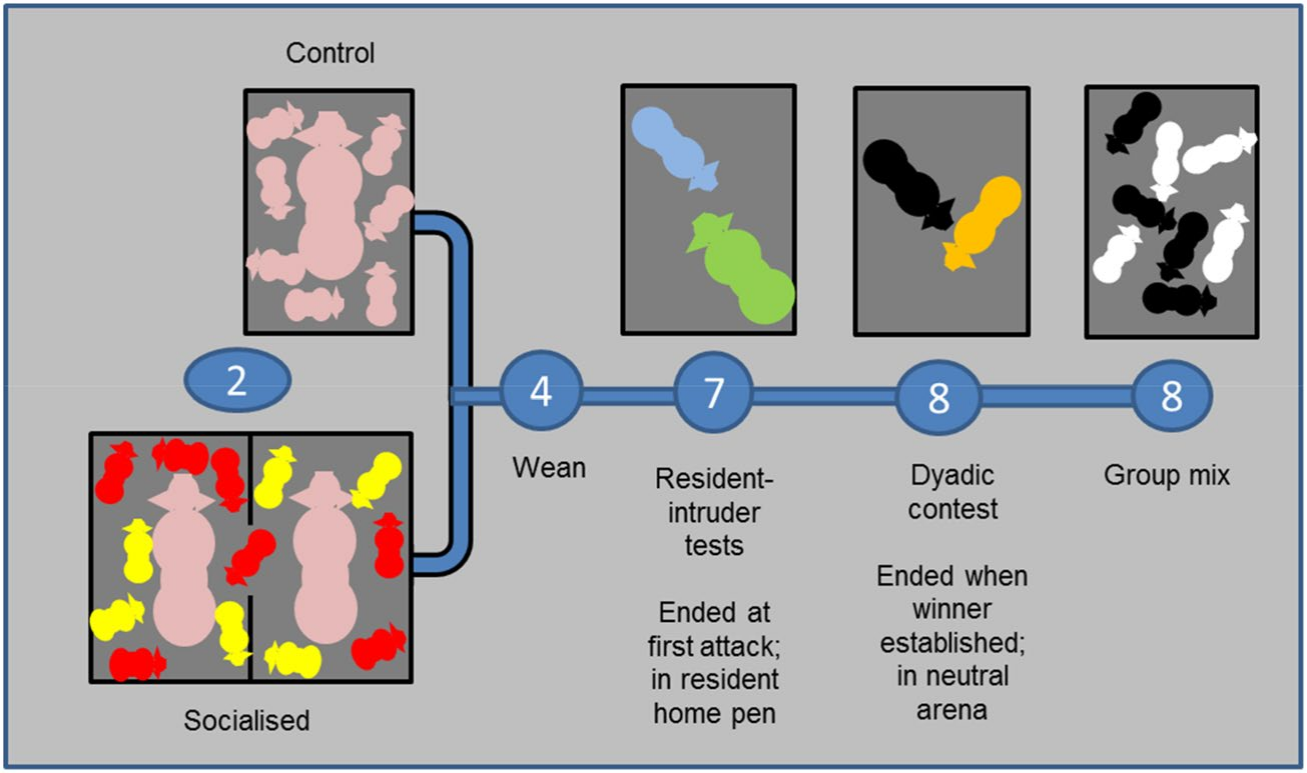
Experimental design. Numbers in circles represent age in weeks.

The experiment comprised of 22 litters born over two farrowing batches (mean litter size 10.8 ± 1.65 piglets). The experiment proceeded in phases; pre-weaning socialisation (week 2 of age); resident-intruder tests (week 7); a dyadic contest (week 8) and group mixing (week 8) (Fig. 1). The farrowing environment conformed to conventional indoor management systems typical of the UK. The farrowing crate housing for a lactating sow and her litter measured 3.15 × 1.50 m in total. It had a solid floor except for a small slatted dunging area at the rear and had a 2.25 × 0.55 m sow crate in the middle of the pen and a 0.65 × 1.50 m heated creep area for the piglets.

#### Pre-weaning socialisation

In each batch, 6 of the 11 litters experienced pre-weaning socialisation (‘socialised’) whilst the remaining 5 litters did not (‘controls’). Litters were selected for socialisation based on two rules; (i) being in adjacent pens and (ii) being born within 48 h of each other. Socialisation was performed by allowing free movement of the piglets but not the sows between farrowing crates from 14 days of age until weaning at 26 days (see Camerlink et al. (2019)^36^ for a detailed description). At weaning, control piglets remained in their litter group and socialised piglets were housed only with their biological littermates in straw-bedded pens allowing 1.1 m^2^/pig. Food and water were provided ad libitum.

#### Tests of aggressiveness and the outcomes of aggressive interactions

The paragraphs that follow describe the use of the resident-intruder test as a measure of aggressiveness in pigs and quantification of the outcomes of aggressive interactions in two social contexts (a dyadic contest and group mixing). All of these scenarios involved interactions between unfamiliar pigs but their conduct and purposes differed. Latency of a resident to attack an inferior intruder in its home pen in the resident-intruder test is a repeatable means of quantifying aggressiveness^37,38^. We used the attack latency to account for aggressiveness in our statistical models that examined the effect of play fighting on skin lesions from the later dyadic contest and group mixing. Hence, this test was performed in the resident’s home pen and was required to progress only until the resident initiated an attack (or other end-points were reached as described below). Attack latency was used in later statistical models as an explanatory variable rather than as a response variable. The effect of play fighting network centrality on the outcome of aggressive interactions was tested using the number of skin lesions from the dyadic contest and group mixing as response variables. The dyadic contest and group mixing both involved introducing unfamiliar pigs into a neutral location with which no pig had prior residency experience. The dyadic contest occurred in an arena that lacked resources such as food and progressed until one member of the dyad lost the encounter (when it did not retaliate to an attack from the opponent), at which point the pigs were returned to their home pen. The group mixing scenario mimicked the combining of social groups (or parts thereof) in commercial pig production. In this highly dynamic context, pigs must establish dominance relationships with several unfamiliar animals in a new home-pen environment that provides essential resources. Furthermore, the group mixing scenario allowed examination of the effect of play fighting network centrality on the number of lesions resulting from establishment of dominance relationships and the number resulting from chronic aggression from maintenance of dominance relationships in the weeks after group mixing.

#### Resident-intruder tests

At 7 weeks of age all pigs acted as residents in two resident-intruder tests on consecutive days with a different intruder on each day. The effect of play fighting experience and socialisation on attack latency has been described by Weller et al. (2019)^16^ and these analyses will not be described further here. However, attack latency as an indicator of aggressiveness was included as a fixed effect in the models described below. A resident was isolated within a familiar section of its home pen and a smaller unfamiliar intruder pig (mean of 76.9 ± 10.24% of the resident’s body weight) was introduced. The latency from first contact until the resident bit the intruder was recorded. The latency between introduction of the intruder and first contact was not analysed as it is affected by the time taken for the resident to notice the arrival of the intruder and to establish that it is unfamiliar. The opponents were separated immediately upon the occurrence of the attack and returned to their respective pen. Tests were ended and the resident received no attack latency time if no aggression was observed within 5 min, if the intruder initiated the attack or if mounting or escape attempts occurred. The mean attack latency from the two resident-intruder tests was used in the models described below.

#### Dyadic contest

At 8 weeks of age all pigs experienced a single dyadic contest as described in detail by Camerlink et al. (2019)^36^. Briefly, pigs encountered an unfamiliar individual in an arena measuring 2.9 × 3.8 m that was novel to both animals. Pigs were paired based on having the same treatment (socialised or control), and were allocated to an opponent to create balanced groups with regard to sex, aggressiveness and body weight difference. The mean body weight difference between contestants was 2.41 ± 2.33 kg (on a mean weight of 22 kg). The pigs were separated and reunited with their litter after (i) a contest loser was established (defined as retreating and failing to engage in aggression for 1 min); (ii) a 20 min time-out period was reached; (iii) pre-defined mounting or escape behaviour thresholds were reached. The number of skin lesions present before and immediately after the contest was counted by a single observer using the method of Turner et al. (2006)^23^.

#### Group mixing

Three days after the dyadic contest, pigs were mixed into new social groups of 12. Pens allowed 0.80 m^2^/pig and had a light straw bedding. All animals on the unit were regrouped following weaning in line with most commercial farms and the group mixing for this study was therefore not additional to routine practice. Pigs had ad libitum access to water and pelleted commercial feed. Groups included both males and females from three or four litters whereby each pig had at least one sibling present. Pigs from two previously socialised litters were not reunited. Skin lesions were counted on the morning before mixing, 24 h after mixing to encompass the period of peak aggression associated with dominance relationship establishment^39^ and 3 weeks post-mixing (at 11 weeks of age) again using the same observer. Three weeks was chosen as the accumulation of fresh lesions returns to a pre-regrouping baseline at around this time^40^ and we assumed that the stability of dominance relationships achieved in the new group approximates that in the old group at this time. At 11 weeks of age, pigs had a mean weight of 43.6 ± 5.1 kg.

### Play fighting behaviour

Play fighting behaviour was recorded for 263 piglets (137 males, 126 females) of which 135 were socialised (69 males, 66 females) and 128 were controls (68 males, 60 females). From 14 days of age until weaning, piglets were digitally video recorded using time-lapse video equipment (Geovision surveillance hardware linked to GV-1480 playback software) for 15 min, 6 times per day for each pen. The continuous observations occurred at hourly intervals for all piglets in the pen from 10:00 am to 15:00 pm on days 14, 16, 19, 21, 24, and 26 after birth which is within the pre-weaning window of time when play is performed most frequently^41^. Sampling at hourly intervals in all pens controlled for the diurnal variation in activity reported in pigs^42^ and follows the sampling strategy from previous work^26,43,44^. The duration of play fighting was short and, for this reason, only frequency was recorded in line with previous work on pigs^26,43,44^. Marking on the backs of piglets enabled the identification of individuals and the amount of time that piglets were in view was similar for all animals. All play fighting interactions were recorded by a single observer, together with the identity of the initiator and whether the recipient responded by engaging in play fighting or not. All piglets in the pen were observed rather than focusing on focal individuals. A new bout of play fighting was recorded if a dyad ceased playing for greater than 3 s. Rapid face-to-face pushing at a recipient was deemed to constitute play fight initiation and play fighting behaviour included elements of pushing with occasional head knocking and biting as also observed by Newberry et al. (1988)^41^ and Brown et al. (2018)^43^. As outlined by Newberry et al. (1988)^41^ and Pellis and Pellis (2017)^6^, pigs use similar targets and tactics in both play fighting and real fighting. Distinguishing between the two is therefore difficult. However, play fighting differs in form from true aggression by lacking escalation through a series of stages of increasing intensity. Such a discrepancy in form has been argued to be one of the defining features of play by Burghardt (2005)^2^. It can also occur alongside other playful actions such as object play and locomotor play and other playful actions such as pivoting and head tossing^12^. Furthermore, bullying behaviour was not seen at the end of an interaction. To confirm that the interactions recorded as playful led to few injuries we estimated the Pearson correlation coefficient between the number of play fights that occurred on the day of socialisation and the log transformed number of skin lesions recorded 24 h following socialisation at week 2. No significant relationship was found in either socialised (r = 0.04, p = 0.633) or control treatments (r = 0.02, p = 0.847).

### Social network analysis

The basic components of SNA are the animals (called nodes) and connections between them (edges; in this case play fighting interactions). Edges can be weighted (by frequency of interactions in this study) or binary (interactions present or absent) (see^45^) (Table 1). The approach can quantify individual network position or emergent properties of relationships at the sub-structural (e.g. clique) or entire network structural level^46^. Social networks of play fighting were reconstructed using the package *igraph* in R studio (version 1.1.442)*.* Networks were constructed based on only successful (i.e. reciprocated) play fighting interactions. Here we focussed on measures of individual centrality in a network commonly quantified in animal studies (degree centrality, eigenvector centrality, betweenness and clustering coefficient; described below). Furthermore, these metrics have been shown in our previous work on fighting networks with older unfamiliar pigs to predict the amount of skin lesions received^31^ and, when scaled to the group level, several of these methods have been applied to study age-dependent changes in aggressive network structure of pigs^47^. These measures of network centrality are described with illustrated examples in Foister et al. (2018)^31^. The individual centrality measures tested the prediction that individual play fighting network position would influence the number of skin lesions from later aggression (Prediction 1). To allow comparison between socialised and control groups in their overall network structure, these measures of individual centrality were converted into group level metrics of centralisation using Freeman's centralisation equation^48^. This approach calculates a global value for the structure of a network by summing the differences in individual centrality scores between the most central animal and all others in the network. The sum is then divided by the theoretical largest sum of differences in any network of equal size to give a value between 0 and 1, where 1 represents a maximally centralised network (see Foister et al. (2018)^31^ for Freeman’s centralisation equation). To test Prediction 2 that play fighting with non-littermates would reduce the number of skin lesions, assortment was calculated at the pen and individual level as described in the statistical analysis section.

**Table 1:** Summary of network traits with respect to whether they were weighted according to the number of interactions between a dyad (weighted) or not (binary) and whether they were directed according to which pig initiated the interaction (directed) or not (undirected). Pen level centralisation metrics quantify the structure of the whole social group (control litter or socialised pair of litters) whilst individual level centrality metrics describe the position of centrality of an individual piglet within a play fighting network.

	Binary (unweighted)	Weighted
**Pen level**		
Edge density	Social connections that occurred, presented as a proportion of the social connections that could have occurred in a network containing *n* individuals	
Degree centralisation	Extent of pen level inequality in the number of social partners each piglet had. High centralisation indicates one/a few piglets had considerably more playmates compared to the remaining group members	Extent of pen level inequality in the number of play interactions each piglet had. High centralisation indicates one/a few piglets had considerably more play interactions compared to the rest of the group
In/out-degree centralisation	Extent of pen level inequality in the number of social partners each piglet received (in-degree) play invitations from, or initiated (out-degree) play invitations with. High centralisation indicates one/a few piglets initiated or received play invitations from many different piglets, in comparison to the rest of the group	Extent of pen level inequality in the number of play interactions each piglet received (in-degree) or initiated (out-degree). High centralisation indicates a/few piglets initiated or received more play invitations, in comparison to the rest of the group
Eigenvector centralisation	Extent of pen level inequality in eigenvector centrality, calculated by the number of social partners a piglet had and whether the social partners were also well-connected (having many play partners)	
Betweenness centralisation	Extent of pen level inequality in betweenness centrality, calculated by the number of shortest paths in which a piglet was present between all other vertices in the pen. High betweenness centralisation indicates that one/few piglets in the pen connected a number of otherwise unconnected piglets	
Clustering coefficient	Proportion of complete triads present in regards to the number of possible triads in a network containing *n* individuals	
**Individual level**		
Degree centrality	Number of play partners a piglet had normalised by the highest number of play partnerships present in the pen. Degree centrality of 1 indicates that the subject was the most central (had the most play partners) in comparison to the remaining group members	Number of play interactions a piglet had normalised by the highest number of play interactions present in the pen. Degree centrality of 1 indicates that the subject was the most central (most play interactions) in comparison to the remaining group members
In/out-degree centrality	Number of play partners a piglet received (in-degree) or initiated (out-degree) play with, normalised by the highest number of received/initiated play partners that occurred in the pen. In/out degree centrality of 1 indicates that the subject initiated/received play invitations with the greatest number of play mates in comparison to the remaining group members	Number of play interactions a piglet received (in-degree) or initiated (out-degree) play with, normalised by the highest number of received/initiated play interactions that occurred in the pen. In/out degree centrality of 1 indicates that the subject initiated/received the most play interactions in comparison to the remaining group members
Eigenvector centrality	Centrality calculated by the number of social partners a piglet had and whether the social partners were also well-connected (having many play partners)	Calculated by the number of play interactions a piglet had and whether the social partners were also well-connected (having many play interactions)
Betweenness centrality	Calculated by the number of shortest paths in which a piglet was present between all other vertices in the pen. High betweenness centrality indicates that the piglet connected a number of otherwise unconnected piglets	
Clustering coefficient	Triads that a piglet was part of, presented as a proportion of the triads that could have formed based upon the number of individuals the piglet played with	

**Figure 2:**
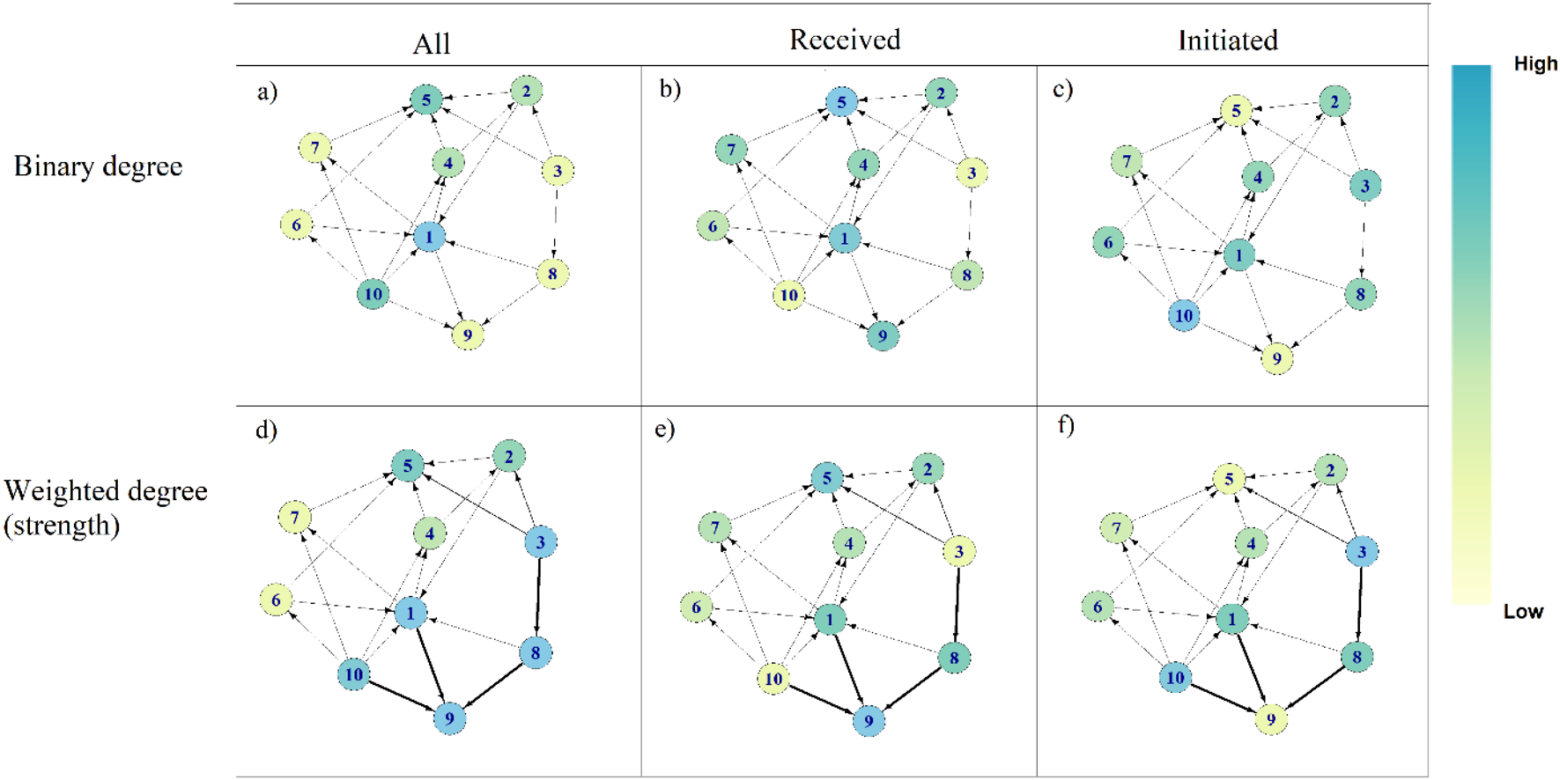
A hypothetical network showing the effect of directed versus undirected degree centrality and of weighting degree according to the number of interactions a pig was involved in (known as ‘strength’). Pig 10 has moderate degree centrality (**a**), low in-degree centrality as indicated by interactions received (**b**) but high out-degree centrality as indicated by interactions initiated (**c**). Pig 10 also has high strength (**d**) but has low strength according to interactions received (**e**) and high strength from interactions initiated (**f**).

**Figure 3:**
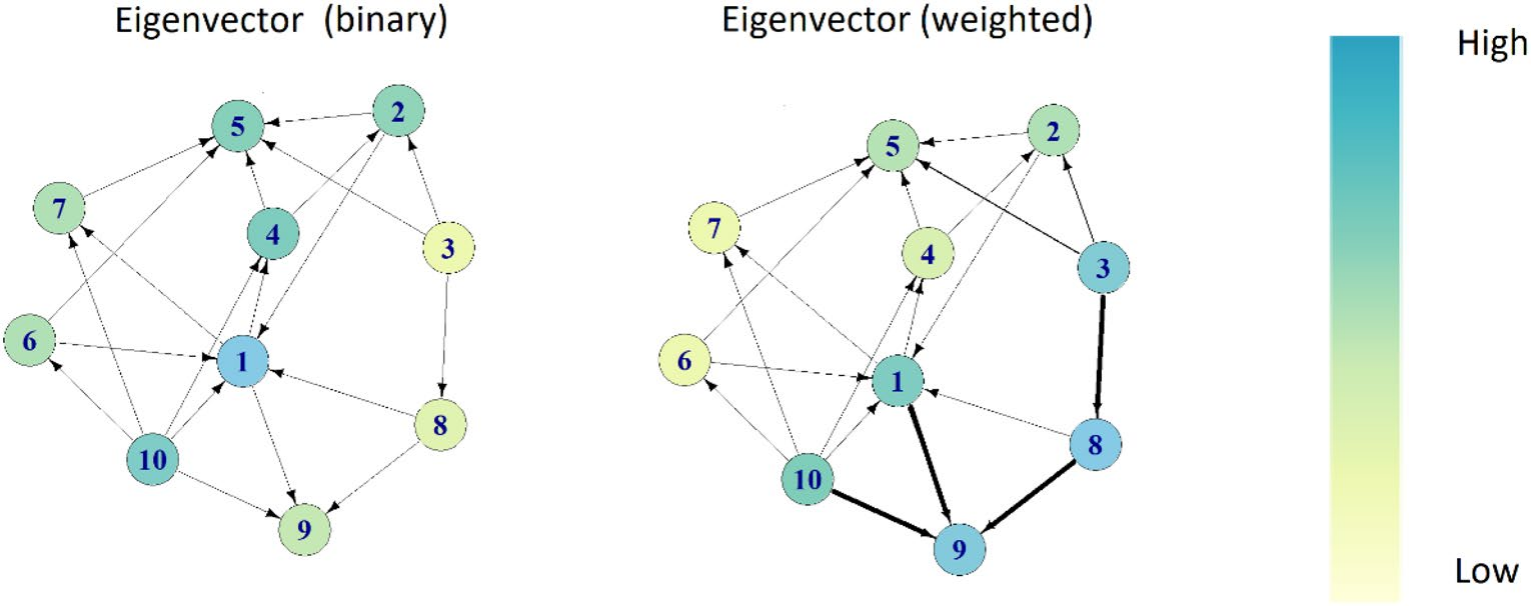
A hypothetical network showing the effect of weighting eigenvector centrality according to the number of interactions a pig engaged in. For example, pig 3 has low eigenvector centrality when it is unweighted but a high centrality when weighted according to the number of interactions between dyads.

We expected that network measures would change substantially in response to whether information on the direction and frequency of interactions was included in their calculation and hence would affect the relationship with subsequent skin lesions. For this reason, a number of network traits (defined in Table 1 and described below) were (i) directed according to which pig initiated the interaction or were left undirected and (ii) weighted according to the number of interactions between a dyad (weighted) or unweighted (binary). For example, at the individual level degree quantifies the number of animals a pig interacted with. Figure 2 uses a hypothetical network to illustrate the effect on degree centrality of directing and weighting the number of interactions a pig had. Eigenvector centrality quantifies the number of social partners a piglet had and whether these social partners also had many social partners. However when eigenvector centrality is weighted, it reflects whether the social partners of a pig had many play fighting interactions. Figure 3 illustrates how eigenvector centrality can change substantially when it is weighted by the frequency of interactions. In our study we, therefore, considered the effect of directing and weighting network traits. A correlation matrix between the different variants of each network measure was used to remove traits that were correlated at r > 0.8.

#### Edge density and in- and out-degree centrality

These are basic measures of connectivity. Edge density is a parameter of the whole group-level network structure (pen level centralisation) and describes the amount of possible interactions between dyads of animals actually observed as a proportion of all possible interactions^47^. As such, a high edge density would occur in a group in which most animals interacted with most others at least once. Degree was used as both an individual animal and pen level metric and both undirected and directed degree were calculated. Directed degree indicates that the number of group members the subject pig received play fight interactions from (in-degree) was calculated separately from the number of group members the subject initiated play with (out-degree) (Table 1). As well as absolute number of play fighting partners (binary degree), degree was also weighted by the frequency of play fighting interactions between partners (known as ‘strength’). At the pen level degree describes to what extent the individual with most interactions played more than the rest of the animals in the network.

#### Eigenvector centrality

Eigenvector centrality is calculated from the number of social partners a subject has and whether the social partners are also well-connected. As such, an individual may achieve high eigenvector centrality due to play fighting with many partners (high degree), by playing with partners who themselves had many play partners, or a combination of both^45,49^. Thus eigenvector centrality accounts for both the quantity of play partners an individual has and how well connected these partners are to others in the network. In the present study both directed and undirected eigenvector were calculated. These were either unweighted or weighted according to the frequency of play interactions between partners to account for the potential that playing with a central individual many times may be more beneficial for the subject than playing with it less often. At the pen level, eigenvector centralisation describes the inequality between piglets in the number and experience of their play fighting partners.

#### Betweenness centrality

Betweenness centrality measures the number of shortest play fighting paths between every pair of group members in the network that pass through a particular individual. In a network graph, animals with high betweenness appear to connect others that do not directly interact^33^. Undirected binary betweenness was used in this study such that betweenness was calculated based only on the presence or absence of interactions between each dyad and irrespective of which animal of a pair initiated the interaction. As a pen level metric, high betweenness centralisation occurs where sub-groups within a pen interact only indirectly through a small number of intermediary animals.

#### Clustering coefficient

Clustering coefficient describes the extent to which two play fighting partners of an animal also directly played together to form a triad of animals that directly interacted^47^. In short, it quantifies (from 0–1) how close a subject’s partners are to forming a fully connected clique. Therefore, it describes the density of play interactions in the part of a network local to a focal individual and captures the ease with which a subject can influence its local cluster of group members^50^. Here undirected binary clustering coefficient was calculated. At a pen level, clustering coefficient reflects the number of triads which interacted as a proportion of the potential total number of triads.

### Statistical analysis

To obtain the number of skin lesions due to aggression during the tests, skin lesion counts recorded immediately before the dyadic contest were subtracted from those recorded afterwards and the number recorded immediately before group mixing was subtracted from the number recorded 24 h post-mixing. The count of skin lesions 3 weeks after group mixing was positively skewed and was log transformed to satisfy the assumption of normality. Statistical analyses were performed in R version 3.5.1.

#### Pen level centralisation

Differences in group level network structure (edge density, degree, eigenvector, betweenness, clustering coefficient) between socialised and control groups were examined using Wilcoxon rank sum tests. To study the extent of non-random integration between the litters that comprised a socialised pair, the assortment value (from -1 (all play occurred with non-littermates) to + 1 (all play occurred with littermates)) for each of the six socialised groups was calculated separately using the R package assortnet and the function assortment.discrete^51^. As advocated by Shizuka and Farine (2016)^52^, interactions between animals were permuted 5,000 times and the likelihood of the observed assortment value within the group occurring by chance was tested by calculating the number of times the observed network assortment value was greater than the values derived from the permuted networks, divided by the number of permutations. Thus, an observed assortment value would be significant at p < 0.05 if it was greater than the assortment of more than 95% of the permuted networks. This value was deducted from 1 where the observed network had a negative assortment value (indicating that piglets in socialised pens preferred to play with non-littermates), in order to provide a p-value^53^. To test whether, at a population level, piglets significantly preferred to play fight with their own littermates as opposed to non-littermates, the total number of play interactions was compared to the number of play interactions that occurred between littermates using a binomial test.

#### Individual level centrality

Differences between socialised and control pens in individual-level centrality traits were assessed using Wilcoxon rank sum tests. To test the effect of individual network centrality (degree, eigenvector, betweenness, clustering coefficient) on skin lesions, separate linear mixed models (LMM) were used (R package lmer using lmer function^54^) for each of three response variables; the number of skin lesions resulting from the dyadic contest at week 8, those accumulated within 24 h of group mixing at week 8 and the number of new lesions present 3 weeks after mixing at week 11. The models firstly excluded network traits but identified the systematic effects that affected these lesion outcomes. All models included sex, socialisation treatment (socialised or control) and attack latency in the resident-intruder test as a metric of individual aggressiveness as fixed effects and pen (litter or socialised pair of litters) nested within farrowing batch as a random effect. Additionally, to predict lesions resulting from the dyadic contest, the weight difference in kg between contestants was entered as a fixed effect and the dyad identity was used as a random effect. To examine skin lesions recorded at 24 h and 3 weeks after group mixing, the number of lesions resulting from the dyadic contest and the body weight of the pig were included as additional fixed effects and the group mix pen identity was included as an additional random effect. Terms found not to significantly affect lesions at p < 0.1 were stepwise removed from the models. Next, all network traits described in Table 1 were entered into a stepwise regression using the R package MASS and the function stepAIC^55^. The stepwise regression was carried out to identify the model of best fit comprising the minimal set of statistically significant network trait predictors of skin lesions, together with collinearity checks (using variance inflation factor). Model fit statistics were assessed based on the AIC. Finally, informative network traits identified in the stepwise regression were then entered as fixed effects into a full linear mixed model to obtain full model statistics alongside the additional fixed and random systematic effects as specified above, and with appropriate interaction terms appropriate to the hypotheses, such as the interaction between network traits and socialisation treatment. The improvement in model fit achieved by including the identified network traits was then assessed using the log-likelihood ratio test between the models with and without the network traits.

At the individual pig level, assortment was calculated as the proportion of a piglet’s play fighting partners that were littermates. To examine the effect on skin lesions of socialised piglets choosing to play fight with littermates or non-littermates, the same modelling approach as described above was repeated only for socialised piglets in which the previously described network traits were replaced with weighted assortment (directed and undirected). Weighted assortment quantified the preference to play fight with littermates over non-littermates whilst accounting for the frequency of interactions with each play partner. Directed assortment separately calculated the assortment value based on play fighting interactions that were initiated or received.

## Results

The effects of age and sex on play fighting have been described by Weller et al. (2019)^16^ together with the repeatability of behavioural expression and the effects of socialisation on the frequency of play behaviour.

### Effect of socialisation on network properties

#### Group-level play fighting network structure in socialised and control groups

##### Edge density

Socialised groups had significantly lower edge density than control groups (socialised median 0.31; control median 0.55, p = 0.015; Table 2). Therefore, in the larger group size of the socialised pens, a smaller proportion of dyads engaged in play fighting with one another and the network appeared to be sparser.

**Table 2:** Median (with inter-quartile range in parentheses) of pen level network centralisation metrics (binary and weighted) for socialised and control (non-socialised) piglet groups. Wilcoxon rank sum test (critical value presented under W, where n1 = 6/n2 = 10). The sample sizes refer to 6 socialised pens each with 2 litters and 10 control pens each with a single litter. P values < 0.05 are highlighted in bold. Betweenness centralisation and clustering coefficient were not assessed for weighted networks.

	Binary				Weighted			
	Socialised	Control	*W*	P	Socialised	Control	*W*	*P*
Edge density	0.31 (0.21–0.46)	0.55 (0.45–0.78)	28	**0.015**	0.49 (0.30–1.06)	1.19 (0.73–1.99)	32	**0.045**
Degree centralisation	0.22 (0.19–0.24)	0.21 (0.19–0.29)	51	1.000	0.86 (0.49–1.41)	1.06 (0.83–1.68)	44	0.481
In-degree centralisation	0.21 (0.15–0.29)	0.23 (0.29–0.29)	44	0.481	0.60 (0.42–1.26)	1.18 (0.78–1.48)	38	0.175
Out-degree centralisation	0.27 (0.24–0.33)	0.34 (0.22–0.46)	41	0.303	1.14 (0.59–1.59)	1.42 (1.07–1.70)	38	0.175
Eigenvector centralisation	0.48 (0.38–0.59)	0.29 (0.18–0.39)	74	**0.015**	0.74 (0.65–0.77)	0.56 (0.45–0.65)	75	**0.011**
Betweeness centralisation	0.10 (0.06–0.14)	0.13 (0.04–0.15)	48	0.786	–	–		
Clustering coefficient	0.58 (0.40–0.59)	0.82 (0.66–0.94)	29	**0.020**	–	–		

##### Degree centralisation

When expressed as a pen-level network property, degree centralisation describes whether certain individuals play fight more than the rest of the animals in the network. There was no significant difference in degree centralisation between socialised and control pens when interactions were weighted according to the frequency of play fighting between each dyad (i.e. strength; Table 2). Piglets with a disproportionately high number of play fighting partners and interactions in comparison to the rest of the pen therefore existed in both types of groups or, conversely, both types of groups lacked pigs with extreme degree centrality. This was also the case when only unique interactions were considered (interactions between a dyad were either present or absent) or when direction was accounted for (Table 2).

##### Eigenvector centralisation

Socialised networks had significantly higher eigenvector centralisation than control pens (socialised median 0.48; control 0.29, p = 0.015; Table 2). This indicates that there was inequality in the distribution of play fighting within socialised groups whereby certain piglets had many play partners and these partners themselves tended to also have multiple play partners.

##### Betweenness centralisation

There was no significant difference in pen level betweenness centralisation between socialisation treatments. Despite socialised groups containing two litters, play fighting interactions between the two litters were not dependent upon a few individuals acting as a bridge between the litters, but interactions were evenly distributed throughout both litters.

##### Clustering coefficient centralisation

Socialised pens had a significantly lower clustering coefficient than control pens (socialised median 0.57; control median 0.82, p = 0.020; Table 2). This indicates that in socialised pens a lower proportion of potential interacting triads formed than in the control pens.

#### Individual level centrality in socialised and control groups

Spearman rho’s analysis showed that binary and weighted network measures were highly correlated, which suggests that individual centrality was not significantly different with the inclusion of multiple interactions. As a result weighted network traits were dropped from further analysis. Table 3 shows the median individual centrality of piglets in socialised and control treatments. Piglets in socialised pens showed a significantly lower degree centrality, eigenvector centrality and clustering coefficient (p = 0.001 to 0.008). This indicates that socialised pigs showed greater equality in their number of play fighting partners and how well these partners were connected, but that fewer of their partners directly interacted to form triads of connected animals within the local part of a pig’s network. Piglet sex had a strong effect on all measures of centrality whereby males performed more play fights, had more play fighting partners and had higher centrality in all measures apart from clustering coefficient (p = 0.07 to p < 0.001).

**Table 3:** Median (and inter-quartile range in parenthesis) of individual pig network centrality measures and skin lesion counts according to socialisation treatment. Wilcoxon rank sum test (critical value presented under W, where n1 = 6/n2 = 10). The sample sizes refer to 6 socialised pens each with 2 litters and 10 control pens each with a single litter. P values < 0.05 are highlighted in bold.

	Socialised	Control	*W*	*P*
Number of play fights	23.5 (12.6–38.0)	21.6 (15.1–32.2)	50	0.956
Number of unique play partners	10.2 (6.8–12.3)	6.7 (5.9–8.3)	67	0.093
**Binary network traits**				
Degree centrality (initiated and received play interactions with unique play partners)	0.48 (0.32–2.07)	0.75 (0.65–0.95)	26	**0.008**
In-degree centrality (receipt of play interactions from unique play partners)	0.31 (0.21–0.46)	0.55 (0.45–0.78)	28	**0.015**
Eigenvector	0.16 (0.15–0.18)	0.27 (0.25–0.30)	21	**0.001**
Betweenness	0.025 (0.020–0.041)	0.029 (0.007–0.039)	51	1.000
Clustering coefficient	0.60 (0.45–0.66)	0.86 (0.69–0.95)	26	**0.008**
**Skin lesion traits (count)**				
Dyadic contest lesions	40.4 (35.7–45.0)	40.9 (26.4–54.5)	51	1.000
24 h post-group mixing	66.9 (63.5–109.6)	72.4 (45.1–116.1)	52	0.957
3 weeks post-group mixing	20.2 (13.2–24.9)	20.1 (16.5–28.8)	46	0.626

#### Assortment of piglets within socialised groups

**Figure 4:**
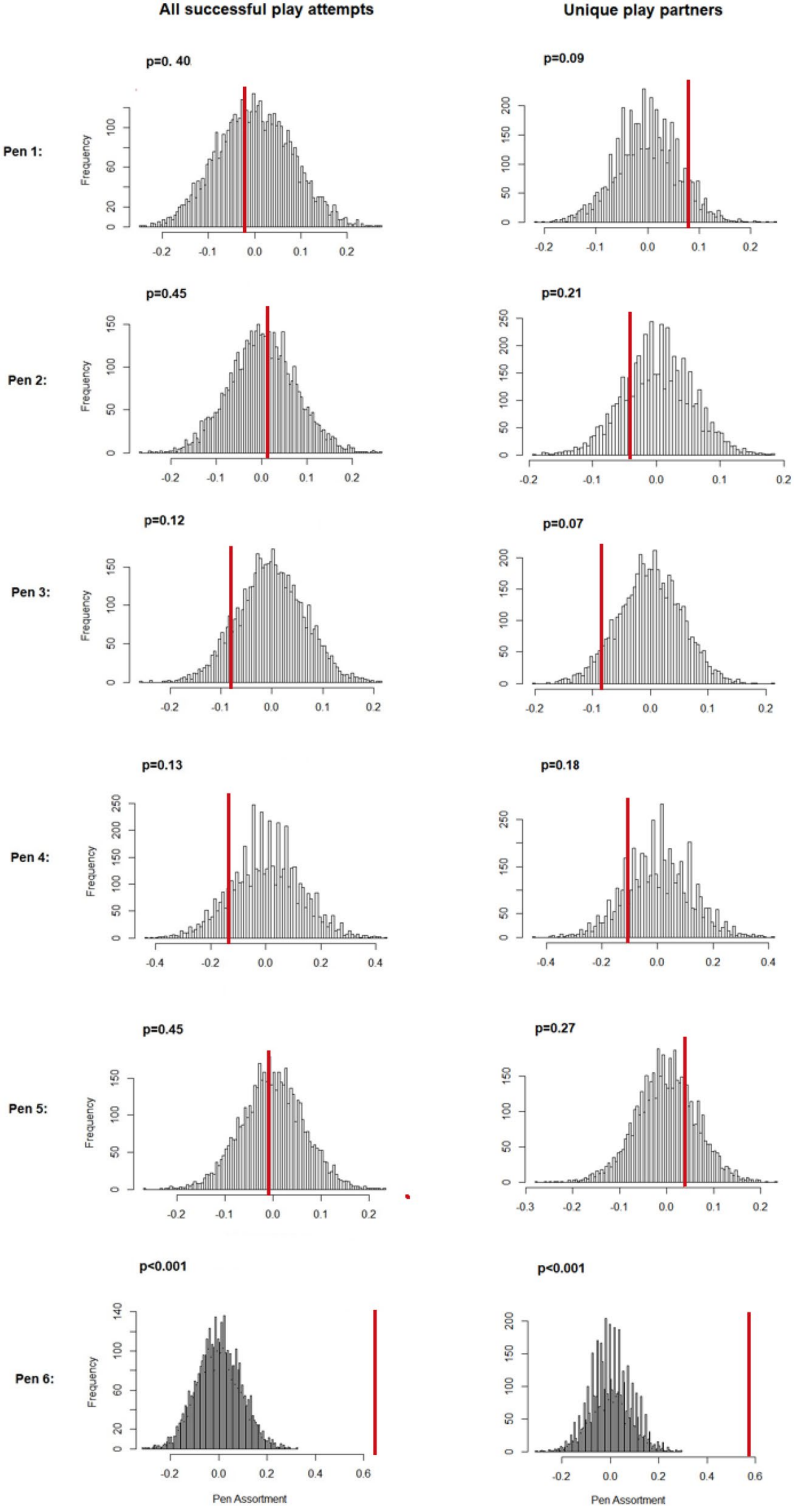
Assortment values for permuted networks for each pen of socialised piglets. The red line on each histogram provides the assortment of the observed pen. P values indicate whether the observed assortment value differed from random.

**Figure 5:**
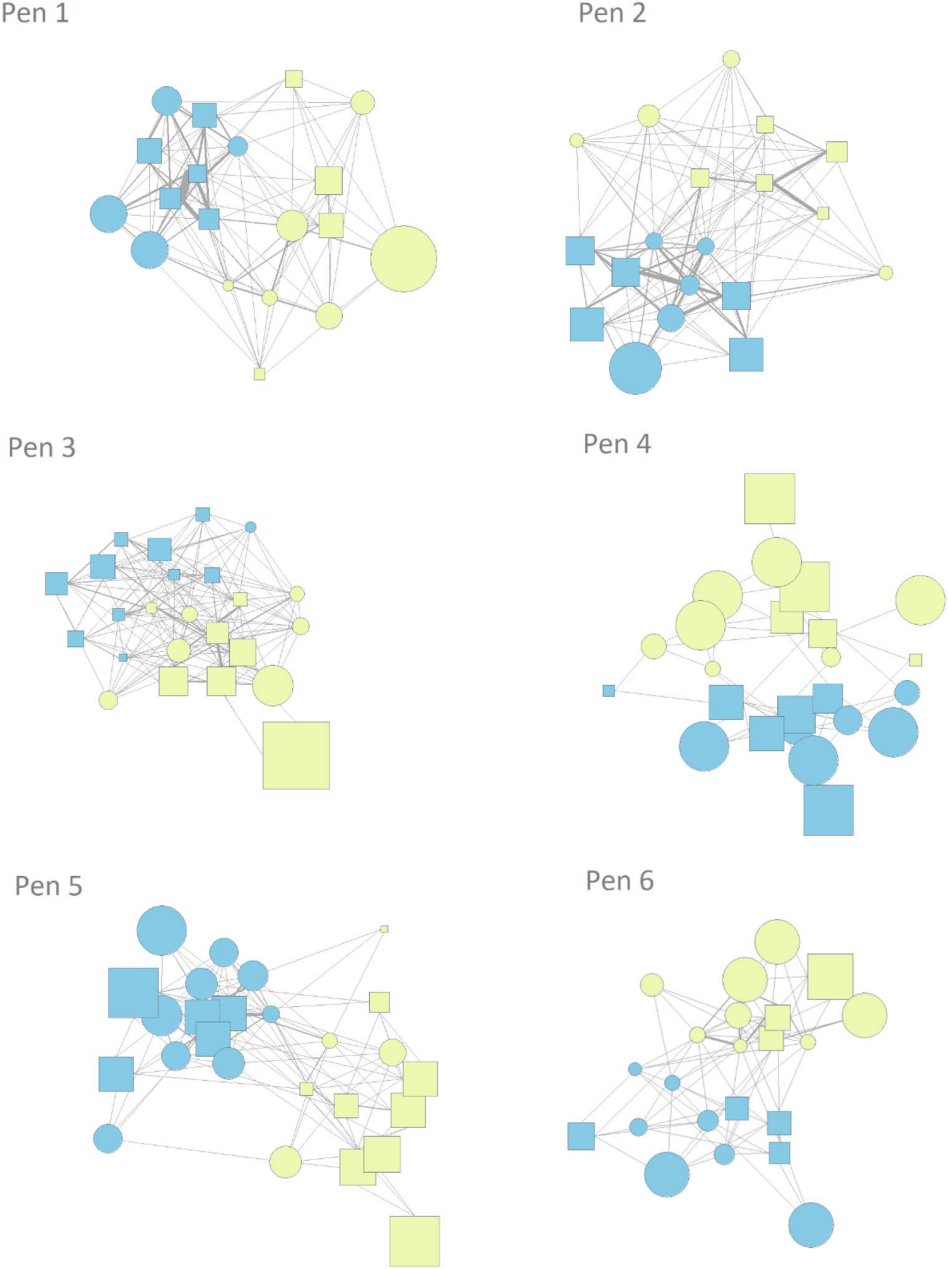
Network images of the six socialised groups. Vertex colour indicates litter membership, vertex shape reflects sex (square = male, circle = female) and vertex size reflects the proportion of play mates that belonged to their own litter (large size indicates the piglet interacted predominantly with littermates). Edge width indicates the frequency of interactions.

At the pen level, only one out of the six socialised pens (Pen 6) showed significant positive assortment, indicating that piglets from the same litter play fought together more often than would be expected at random (p < 0.001; Fig. 4). This was evident in both the weighted and binary cases. Figure 5 shows the interaction of socialised piglets with littermate and non-littermate play fighting partners in each of the six groups. Piglets in Pen 1 also showed a non-significant tendency (p = 0.09) to choose littermates as play fighting partners. Interestingly, Pen 3 showed a non-significant tendency (p = 0.07) towards negative assortment indicating that there were more play fighting interactions between piglets from different litters than would be expected at random. The remaining three pens had assortment values close to zero, indicating no significant preference to play fight with littermates or non-littermates. At the individual level, the majority of piglets preferred to initiate play fights with their littermates (median proportion of invitations to littermates = 0.80, inter-quartile range = 0.30), which a binomial test revealed was higher than expected by chance (p < 0.001).

### Effect of individual network centrality on skin lesions

The socialisation or control treatment did not affect the number of skin lesions from the dyadic contest or those recorded 24 h or 3 weeks after group mixing (Table 3).

#### Skin lesions from dyadic contests

The number of skin lesions from the dyadic contest was unaffected by the systematic effects or play fighting network traits except in-degree centrality where pigs which received play invitations from many play fighting partners received fewer skin lesions from the dyadic contest (estimate from LMM − 54.8, t = − 2.57, df = 85, p = 0.01).

#### Skin lesions from group mixing

Pigs which attacked quickly in the resident intruder test received more lesions at 24 h (estimate from LMM − 0.138, t = − 1.80, df = 84, p = 0.048) and 3 weeks post-mixing (estimate − 0.002, t = − 3.13, df = 83, p = 0.002). No other systematic effects significantly influenced the number of skin lesions 24 h or 3 weeks after mixing. Furthermore, no play fighting network traits influenced the number of skin lesions 24 h after mixing.

A high degree centrality reduced the number of lesions 3 weeks post-mixing but in interaction with the socialisation or control treatment. This indicated that socialised pigs with high degree centrality received more skin lesions than control pigs with high degree centrality, but the difference was not significant for pigs with low degree centrality (estimate from LMM 0.75, t = 2.57, df 136, p = 0.011).

#### Effect of assortment within socialised groups on skin lesions

Socialised pigs were also analysed separately to estimate the effect of play assortment with littermates as compared to non-littermates on skin lesions. No significant effect of assortment on lesions from the dyadic contest (estimate from LMM 36.14, t = 1.13, df = 93, p = 0.200) or from group mixing were found (24 h post-mixing, estimate 40.59, t = 1.046, df = 45, p = 0.301; 3 weeks post-mixing, estimate 0.01, t = 0.06, df = 62, p = 0.95).

## Discussion

Our first prediction was that pigs that occupy a more central position in a play fighting network experience fewer injuries from dyadic contests and group mixing. The second prediction was that those that engage in play fighting with non-littermates when socialised before weaning experience a similar benefit. We tested these predictions using a species in which play fighting closely replicates the aggressive behaviour of adults. The study showed that socialised groups comprised of two litters of piglets had sparser play fighting networks than control groups composed of a single litter. Additionally, within most of the socialised groups, piglets exhibited no statistically significant preference to play fight with either littermates or non-littermates. Males in either socialised or control groups engaged in more play fights, with more partners and had higher centrality than females. The number of skin lesions resulting from the dyadic contest or group mixing was unaffected by most measures of the centrality of a piglet in its play fighting network or, in socialised pens, the tendency to play fight with littermates or non-littermates. However, pigs which received play fighting invitations from many group mates (in-degree centrality) gained fewer lesions in the dyadic contest. Furthermore, pigs which had many play fighting partners (degree centrality) tended to have fewer lesions 3 weeks after group mixing, but this was affected by the socialisation or control treatment. The results of the present study indicate that, under the social contexts tested here, play fighting network centrality and experience of play fighting with non-littermates has limited impact on the number of skin lesions from later aggressive interactions.

In previously reported research, there has been conflicting evidence as to whether engagement in play fighting at a young age reduces the costs of later aggressive interactions. No association between play and later aggression has been found in Syrian golden hamsters (*Mesocricetus auratus*)^13^ and meerkats (*Suricata suricatta*)^14^ whilst an association has been found in rats (*Rattus norvegicus domestica*)^56^, yellow-bellied marmots (*Marmota flaviventris*)^15^ and domestic pigs (*Sus scrofa domesticus*)^16^. This conflict also exists between studies in which animal physiology or the physical or social environment have been manipulated to promote contrasting levels of play fighting (e.g. no association between play and later aggression reported in rats^57^ versus an association reported in domestic pigs^26,58^).

Here the novel application of social network analysis quantified whether play fighting centrality affected the costs of subsequent aggressive interactions with unfamiliar animals. Social network analysis avoids assumptions that dyads interact independently of their wider social group and that partner play experience is irrelevant. To date there has been a striking lack of use of social network analysis to quantify animal social play networks, although this work is now beginning (e.g. pigtail macaques (*Macaca nemestrina*)^59^, African lions (*Panthera leo*)^60^, Japanese macaques (*Macaca fuscata*)^61^, chimpanzees (*Pan troglodytes*)^62^, brown capuchins (*Cebus apella*)^30^, hamadryas baboons (*Papio hamadryas*)^30^, diademed sifaka (*Propithecus diadema*)^30^).

We studied the benefits of play fighting using dyadic contests as this scenario is highly controllable allowing balancing of opponents for sex and weight and avoiding the influence of other animals (e.g.^36,63^). Group mixing was also studied as this is highly relevant for commercial production whereby new social groups are formed at multiple points in the production cycle leading to large numbers of skin lesions (e.g.^23^). Play has also been shown to benefit the integration of adults into social groups in Verreaux’s sifaka (*Propithecus verreauxi*)^64^. For the group mixing, skin lesions were recorded following the acute period of dominance relationship establishment (focussed on the first 24 h in immature pigs^39^) and 3 weeks after group formation to record the occurrence of on-going chronic aggression.

Here we focussed on network centrality traits that have most commonly been used in studies of animal behaviour. The measures were previously used to show that the aggression network structure of a group could predict the number of injuries better than the sum of dyadic interactions (e.g. total duration of fighting that occurred in the group)^31^. The number of skin lesions pigs received from the dyadic contest or group mixing varied greatly. The number of play fight partners, number of play fight interactions and the centrality of piglets within their network also showed large variation. Median eigenvector centrality and betweenness were both low when expressed either as a measure of the group network structure or of the centrality of individual piglets within their network. Low values have previously been reported with regard to individual centrality or pen-level network centralisation of true aggressive behaviour in pigs^31,47^. In the current study, little inequality therefore existed in the distribution of play fighting as measured by eigenvector or betweenness. Specifically the low eigenvector value suggests that most piglets engaged in play fights with piglets who themselves were not highly experienced in play. The low betweenness indicates that play fighting was not focussed within distinct subgroups that were linked by specific ‘gatekeeper’ animals. Clustering coefficient was higher suggesting that the direct play partners of a pig also tended to play together.

### Prediction 1: Network centrality will lead to fewer skin lesions

The number of skin lesions pigs received from the dyadic contest or that were present 24 h or 3 weeks following group mixing was largely independent of their play fighting network centrality. Pigs with a high in-degree received fewer lesions from the dyadic contest and those with high undirected degree centrality tended to receive fewer lesions 3 weeks after group mixing. This in line with evidence that a pre-weaning environment that promoted play fighting between piglets resulted in more short-term but less long-term aggression when regrouped at weaning^26^. Similarly a pre-weaning environment that stimulated more play also reduced aggression in a later food competition test between familiar group members^58^. Therefore, there appears to be a benefit of having many play fighting partners for the ability of pigs to avoid or resolve later conflict.

Refinements to our study could benefit future work in this area. Play fighting bouts were typically of short duration and we only weighted networks according to the frequency of interactions. Weighting them according to duration may have resulted in different effects of play fighting on skin lesions. Our outcome measure of skin lesions reflects the number of successful bites. We are unable to make any assumptions about the effect of play fighting experience on the skill with which the opponent was able to inflict injuries, nor the skill of the subject in defending against injury. It is, therefore, possible that play fighting influences not only the actual number of bites received but also the proportion of attempted bites that cause injury and this should be examined. In our study, socialised pigs faced socialised opponents and control pigs faced control opponents, but no attempt was made to stage interactions between animals with respect to their play fighting experience. Our finding that most measures of play fighting centrality did not affect the number of lesions could also reflect the susceptibility of play fighting effects to being overshadowed by subsequent social experience as reported previously in rats^65,66^. In this study the opportunities for new social experiences between experiencing play fighting and later aggressive situations were few apart from the attack latency tests. Whilst this may have minimised the likelihood that intervening experiences would mask the benefits of play, the animals were still pre-pubertal and potentially too young when the dyadic contests and regrouping took place. It has previously been argued that social play has an immediate benefit in preparing young to integrate into a larger communal social group in the first weeks of life (spotted hyaenas (*Crocuta crocuta*)^67^). Such integration also occurs in wild pigs^68,69^. However, intense aggressive interactions are unlikely to be frequently experienced in wild pigs before puberty and the young age at which we staged aggressive interactions may have reduced their relevance to the wild context even though the pigs we studied were capable of a complex and damaging aggressive behavioural repertoire.

It has been argued by Bekoff (2001)^10^ that the immediacy of the benefits of play may differ according to sex and indeed sexual dimorphism in the effects of play experience in pre-pubertal pigs has been described^16^. We found clear evidence of greater engagement in play fighting and higher individual centrality in males in this study. Greater engagement of males in play fighting may have been selected for due to the severity with which wild adult boars compete with each other during the breeding season for access to females^16^. In some species, adult male-male competition is not associated with heightened male engagement in play fighting^70,71^. However, in a range of other ungulate species where competition between adult males exceeds that between females, males engage in more social play, in some cases including forms of play fighting (e.g. Siberian ibex (*Capra ibex sybirica*)^72^, bighorn sheep (*Ovis canadensis*)^73^, domestic lambs (*Ovis aries*)^74^, domestic beef calves (*Bos indicus*)^75^ and horses (*Eguus caballus*)^76,77^). It is, therefore, possible that the benefits of a central play fighting network position on later conflict resolution become more apparent in older pigs and that the timing of this apparent benefit may be sex-dependent and reflect differences in social strategy of male and female wild pigs. However, substantially higher dyadic contest aggressiveness has been described^25^ in males at the young age studied here and an effect of play fighting experience on male-male aggression may therefore have been expected. For ethical and practical reasons, staging dyadic contests and group mixing between older, and especially sexually mature pigs, was not undertaken. However, clearly more effort is required to determine the age at which the benefits of play fighting are greatest.

It is also possible that the quantity and quality of play fighting interactions was constrained by the pre-weaning accommodation. Piglets were housed in pens with conventional farrowing crates. The provision of a larger pre-weaning environment in which greater interaction with the sow was possible has been found to increase some aspects of play in piglets^26^. Although we recorded play fighting observations during the age window when piglets show their peak in play^41^ the sampling strategy and sample size may have been too small to overcome any constraints on play fighting imposed by the farrowing crate environment.

### Prediction 2: Play fighting with non-littermates will lead to fewer skin lesions

Socialisation at 2 weeks of age attempted to replicate the early-life integration of litters seen naturally around this time^68,69^. We expected that the opportunity to engage with new play fighting partners and to have greater choice of partners in terms of their individual attributes such as weight would enhance the quality of the play experience. Previous work suggests that animals are selective in their choice of play partners. For example, piglets have been found to prefer partners of the same sex, weight and litter^78^. The present study did not analyse the attributes of play fighting partners (e.g. their weight) but these attributes could influence the benefits derived from play fighting. The effect of play partner attributes on later costs from aggressive interactions could be considered in future work as a further refinement to the calculation of centrality metrics.

In the present study, doubling the number of potential play fighting partners only increased the actual number of partners by a factor of 1.5 in socialised groups and no effect was apparent in the total number of play fights that each pig engaged in. Consequently, fewer of the potential connections between animals actually existed in the socialised networks and these appeared to be sparser. This was evident in the lower edge density and clustering coefficient of socialised groups and the lower degree centrality and clustering coefficient of individual piglets in the socialised pens. From this it would appear that there is a ceiling to the number of play fight interactions a piglet is willing to engage in during the period from 2 to 4 weeks of age. This is in line with earlier work that found that play frequency was similar in piglets of single-housed sows as compared to sows housed in groups of 6–12 where the number of potential play partners was much larger^19^. It is possible that the larger group size in the socialised pens allowed piglets to engage more selectively with partners with specific attributes or experience. Socialised animals may, therefore, have achieved the same benefits from play fighting with a smaller proportion of their group members. However, socialisation did create structural differences in play fighting networks at the group level. The higher eigenvector centralisation of socialised groups suggests that there was less equality in the distribution of play fighting within these groups and certain piglets had many play partners which themselves were well connected to others.

In three out of the six socialised groups piglets interacted at random with littermates versus non-littermates. In one pen piglets showed a statistical tendency to play fight with non-littermates in preference to littermates. In these four pens, piglets were not limited to engaging in play fighting with littermate partners who they had established relationships with during the first two weeks of life. In the remaining two pens there was evidence that piglets preferentially assorted with littermates over non-littermates. Large litter differences in piglet play fighting have been described^43^ and the differences in assortment between pens in the present study may partially reflect such variation. In the population as a whole, a statistically significant preference to choose littermate play fighting partners on 80% of occasions does suggest that piglets were conservative in their willingness to play with non-littermates. This agrees with evidence that weaned pigs prefer to engage in social play with littermates^78^. Despite the fact that most socialised piglets engaged in play fighting at least once with non-littermates and that there was substantial variation in the proportion of play interactions that occurred with non-littermates, no effect of this assortment on subsequent skin lesions was found. Evidence that individual piglets had a significant preference to play with littermates, whilst at the group level this positive preference was only seen in two pens (one at p = 0.09), highlights the value of considering social interactions at both individual and group levels.

Interestingly, degree and whether or not socialisation had occurred interacted to affect the number of skin lesions 3 weeks after group mixing. Socialised pigs with high degree centrality received more skin lesions than control pigs with high degree centrality. It is unclear why socialised pigs would benefit less from playing with a high proportion of their penmates when the early social environment was designed to more closely replicate natural conditions than a standard commercial environment. Commercial pigs exhibit the behavioural repertoire of wilds pigs when in a natural environment^79^, meaning that the socialisation performed probably retained ecological relevance for these animals. Although there were structural differences in the play fighting networks of socialised and control pens as discussed above, no other individual-level centrality measure affected the count of skin lesions at 3 weeks post-regrouping. It, therefore, seems unlikely that the smaller benefit of a high degree in the socialised pigs was due to alterations in the play fighting network structure or specific characteristics of an individual’s position in the network. This deserves further investigation but it appears that the benefits of engaging in play may be sensitive to the environment in which play occurs (Supplementary information).

## Conclusions

We found evidence that frequent engagement with a large number of play fighting partners can reduce subsequent injuries from aggression in a dyadic contest and between familiar group members but, in the latter case, that this benefit may be affected by the early life environment. The position of centrality of a pig in its play fighting network otherwise did not affect subsequent skin lesions, in contrast to Prediction 1. Furthermore, although early life socialisation affected the play network structure at the group level, it did not stimulate an increase in play interactions. Socialised piglets preferred to play fight with littermates although this was highly pen-specific. The extent to which piglets play fought with non-littermates did not affect the costs paid in skin lesions from subsequent aggressive interactions which refutes Prediction 2. Although most measures of network centrality did not appear to affect later aggression, we advocate the use of SNA to complement conventional methods of quantifying play behaviour and for studying the effects of early social group interactions on later life experience.

## Supplementary information

Supplementary Information.

## Data Availability

Data are provided in the supplementary material.
